# Review on Icariin as A Novel Medicaments to Confront Ovarian Cancer: Role of Icariin Against Ovarian Cancer

**DOI:** 10.31661/gmj.v11i.2809

**Published:** 2022-12-31

**Authors:** Sima Mousavi, Solmaz Khastehband, Sahar Poudineh, Maryam Poudineh, Alireza Sarlak, Behnaz Barghgir, Sheida Jamalnia

**Affiliations:** ^1^ Department of Obstetrics and Gynecology, School of Medicine, Ardabil University of Medical Sciences, Ardabil, Iran; ^2^ Department of Educational Management, Islamic Azad University-South Tehran Branch, Tehran, Iran; ^3^ School of Medicine, Mashhad Azad University, Mashhad, Iran; ^4^ School of Medicine, Hamedan University, Hamedan, Iran; ^5^ School of Medicine, Shahrud University, Shahrud, Iran; ^6^ Department of Nursing and Midwifery, Kazeroun Branch, Islamic Azad University, Kazeroun, Iran

**Keywords:** Ovarian Cancer, Icariin, Phytochemical, Apoptosis, Survival

## Abstract

Ovarian cancer is described as one of the most common types of cancer and the leading cause of cancer-related deaths due to the high aggressiveness of this malignancy. However, the current therapeutically strategies failed to confront ovarian cancer or are accompanied by significant adverse effects leading to the recurrence of the disease and/or affecting the quality of life of survivors. On the other hand, ovarian cancer is recognized as a heterogenous disorder that is specified by alteration in a variety of molecular and cellular markers. Thereby, researchers are keen to find a novel therapeutical strategy representing high efficacy and safety, as well as be able to modulate altered biomolecules and signaling pathways. Icariin is a phytoestrogen with desired properties that are suggested for several chronic complications, particularly different types of cancer. The aim of the present study was to reveal the ameliorative characteristics of icariin and then discuss the antitumoral activities of this phytochemical against ovarian cancer with an emphasis on the modified molecular signaling pathways.

## Introduction

Cancer remains one of the leading causes of mortality and morbidity worldwide; as per recent statistics, approximately two million new cases and more than 600,000 deaths are projected to occur in the United States in 2022 [1]. Ovarian cancer is considered the third most common gynecological tumor after cervical and uterine cancers; however, the remarkable aggressivity has made it the leading cause of cancer deaths in women as well as the fourth rank of death among all fatal diseases in women [2][3][4]. Statistical studies state that annually over 240,000 new cases are diagnosed with ovarian cancer, and approximately 380,000 deaths occur per year worldwide [5][6].

World health organization has histologically classified ovarian tumors based on histogenetic principles according to the tumor derivation from coelomic surface epithelial cells, mesenchyme, and germ cells [7]. The majority of malignant ovarian tumors are considered to be epithelial ovarian cancers, which are further divided into mucinous, serous, clear cell, mixed epithelial tumors, transitional cell tumors (known as Brenner tumors), carcinosarcoma, endometrioid, undifferentiated carcinoma, and other histological types [8]. Moreover, some types, such as clear cell and endometrioid carcinomas, have been associated with endometriosis, another gynecological disorder [8][9]. Importantly, this most common type of ovarian cancer, epithelial ovarian cancer, has a 5-year survival rate of 45.6% and could be caused by hormone imbalance during physiological processes such as ovulation and pregnancy, as well as exogenous estrogen and progesterone [10][11][12].

The primary and standard for ovarian cancer treatment broadly includes debulking surgery to no residual disease followed by platinum-based chemotherapy, accompanied by anti-angiogenic agents in a patient who has suboptimal debulked and advanced (stage III-IV) ovarian cancer; however, the outcomes of the disease management are complicated because of different factors [13]. Firstly, ovarian cancer is considered a heterogeneous group of malignancies representing different etiology and molecular biology, even in a similar histological class [14]. Secondly, it is documented that the early symptoms of this type of cancer are occult. Moreover, the identification of the tissue types and whether the tumor is benign or malignant is quite challenging [15]. Notably, the 5-year survival rate for ovarian cancers is reported to be 93% when diagnosed at an early stage but declines to just over 13% when diagnosed at an advanced stage [5]. The occultness of early symptoms, the poor prognosis of the disease in advanced stages, and the fact that about 70% of ovarian cancer diagnoses are made in advanced stages [15] ultimately lead to non-responsiveness to therapeutic strategies and reduced rates of survival to the point where 63% of cases ends in death [15]. In addition to all this, the adverse effects of common treatment methods, such as the invasiveness of surgery and the toxicity of chemotherapy on non-target tissues, which cause infertility [16][17], anemia [18][19], infection [20][21], bleeding [22], insomnia and depression [23][24][25], diarrhea, and constipation [26][27] have led researchers to desire to find an alternative to the standard treatments strongly.

The design and development of novel pharmaceuticals with fewer adverse effects and improved antitumoral activity are considered one of the main strategies to confront the previous insufficiencies of ovarian cancer therapeutic approaches [28][29]. Nevertheless, this solution itself faces defects such as being costly, time-consuming approval processes, and a lack of ability to eliminate all the previous adverse effects. Poly (ADP-ribose) polymerase (PARP) inhibitors, for example, benefits from homologous recombination deficiency, particularly in the carriers of breast cancer gene 1 and 2 (BRCA1/2) mutation [30][31]. Furthermore, aurora kinase inhibitors in certain tumor types, such as epithelial ovarian cancer, have been suggested by extensive recent preclinical studies [32][33]. In addition, the determined mutations (e.g., *ARID1A* mutations) along with aberrant signaling pathways (e.g., phosphatidylinositol 3-kinase [PI3K]/Akt/mTOR pathway) are considered the main characteristics of ovarian clear cell carcinoma and endometrioid ovarian carcinoma proposing further therapeutic targets [34][35][36][37].

Fortunately, herbal compounds and traditional Chinese medicine have been demonstrated in several studies to provide desired features such as antitumor [38], anti-inflammatory [39], antimicrobial [39], antioxidant [40], metabolism regulation [41], antidiabetic [42], antineurodegeneration [43], cardioprotective [44], enhancing the effects of chemotherapy [45], and reducing the destructive adverse effects of pharmaceuticals in non-target healthy tissues [46]. The present study aimed to assess the antitumoral activity of herbal products against ovarian cancer, introduce Icariin, a novel dietary phytochemical with extensive beneficial properties, and finally review its therapeutic performance against ovarian cancer.

## Phytochemicals Target Cancer Cells Survival and Proliferation

It is well known that cancer does not arise due to a single target disruption; however, it involves consecutive genetic and epigenetic changes, all of which lead to a myriad of altered signaling pathways. Hence, full knowledge of the complicated character of cancer still confronts a hard-to-estimate number of challenges [47]. In addition, the involvement of multiple signaling pathways via sequential genetic and epigenetic changes confirms that the proposed therapeutic approach must be capable of modulating the altered factors in addition to representing safety and reasonable adverse effects, not to decrease the quality of life of survivors [29][47][48][49]. Interestingly, phytochemicals, which are abundantly found in the daily diet and are inexpensively available to the public, propose the potential for such a function widely [50][51][52][53][54][55][56].

It is extensively reported that natural compounds are capable of altering key regulators of tumor glycolysis signaling pathways, including glucose transporters, phosphofructokinase, hexokinases, lactate dehydrogenase, and pyruvate kinase and thereby affecting tumor cells' energy sources to restrict their proliferation. Additionally, the synthesis, activation, stabilization, and accumulation of hypoxia-inducible factor 1-a in cancerous cells are affected by phytochemicals via modulation of PI3K/Akt/mTOR and MAPK/ERK signaling pathways [47]. It is documented that phytochemicals can modulate apoptotic and autophagic signaling pathways in cancer, making these compounds promising therapeutic options [57]. Indeed, numerous studies demonstrated that phytochemicals affect cell survival signaling pathways in a pleiotropic and poorly specific approach; however, the modulation of reactive oxygen species (ROS) levels leads to activation of survival or a pro-apoptotic and pro-autophagic mechanism in the targeted tumor cell is common among all of them [58]. The regulatory role of the natural compounds on the crosstalk between apoptosis and autophagic flux could determine the destination of cancerous cells [59].

In addition to this antioxidative property of phytochemicals, these bioactive compounds are capable of targeting the signaling pathway related to toll-like receptor4 (TLR4), a well-known pattern recognition receptor that plays a remarkable role in the host immune system in which its triggering is followed by the secretion of pro-inflammatory cytokines and chemokines and the activation of both innate and adaptive immunity, leading to anti-inflammatory responses and cancer prevention [60]. More importantly, the combined administration of phytochemicals with chemotherapeutics, known as polychemotherapy, could enhance anticancer activity by inhibiting chemoresistance via downregulation of oncogene pathways, including transforming growth factor -β (TGF- β), matrix metalloproteinase (MMP)-2, PI3K/Akt, EMT, NF-κB, and AP-1, augmentation of apoptosis induction in cancer cells, and suppression of cancer metastasis and proliferation [61]. In addition, the effects of selected phytochemicals or their combination on Nrf2 and NF-κB activities represent cancer prevention and therapy properties [62]. Furthermore, the Janus kinase (JAK)/signal transducer and activator of transcription (STAT) signaling pathway, which its aberrant activation leads to tumorigenesis, is suppressed by phytochemicals leading to impeding cancer cell growth [63].

## Phytochemicals Against Ovarian Cancer

Similar to the mentioned content in the previous section about phytochemicals' potential therapeutic role through modulation of several signaling pathways in all types of cancers, many studies have stated these beneficial effects in ovarian cancer. Resveratrol, for instance, is recognized as a preventive and therapeutic agent for ovarian cancer since it is capable of targeting a variety of oncogenic and oncosuppressive pathways, including cancerous cell proliferation, metastasis, autophagy, apoptosis, and sensitization [64]. Furthermore, quercetin, a well-known phytoestrogen, is reported to be able to attenuate metastatic features of human ovarian cancer cells by inactivation of PI3k/Akt, Ras/Raf pathways, and epidermal growth factor receptor expression, which are involved in ovarian tumor cell survival and proliferation along with modulating the levels of migration and adhesion signaling molecules such as occludin, claudin-4, and claudin-11 [65]. Similarly, the decrement in antiapoptotic molecules (e.g., Bcl-2 and Bcl-xL) but the increment in pro-apoptotic molecules (e.g., Bad, Bax, Bid, caspase-3, and caspase-9) revealed that quercetin could inhibit the growth and survival of metastatic ovarian cancer cells [66]. Also, such a function has been reported following the interplay of quercetin and microRNAs [67]. The regulation of ovarian cancer cells carcinogenesis through modulation of the Wnt/β-catenin signaling pathway [68], suppression of ovarian cancer cells metastasis via affecting the JAK/STAT3 pathway [69], and disrupting tumor proliferation, growth, and angiogenesis via downregulation of PI3K/Akt and MEK/ERK1/2 axes are desired properties of curcumin [70]. Fortunately, the findings are not limited to the mentioned examples, and many further studies have provided similar reports regarding other phytochemicals [71][72][73].

## Icariin, A Novel Phytochemical with Favored Medicament Properties

The dried leaf of *Epimedium*, an herbaceous plant belonging to the family of *Berberidaceae*, is known as Epimedii herba. This plant is abundantly found in different parts of Asia as well as Europe [74]. *E. herba* has been prescribed for over 2000 years in Eastern Asia countries by traditional Chinese medicine practitioners for its therapeutic functions [75]. Chronic disorders such as female sterility, chronic bronchitis, general edema, leucopenia, kidney disorders, viral myocarditis, and hypertension are among the most important conditions that *E. herba* can provide a beneficial alleviating role [74][75]. The therapeutic properties of *E. herba* are attributed to bioactive compounds, including flavonoids, terpenoids, and other chemicals such as steroids, acids, lignans, alkaloids, and anthraquinones [76]. It has been determined that there are 53 different flavonoids in this plant, including baohuoside I [76], ginkgetin [77], quercetin [78], robinetin [75], apigenin [75], luteolin [79], hyperin [77], and icariin [80].

Icariin, 2- (4′- methoxylphenyl)- 3- rhamnosido- 5- hydroxyl- 7- glucosido- 8- (3′- methyl- 2-butylenyl)-4- chromanone, is a well-known pentenylated flavonoid glycoside monomer derived from *E. herba* [80]. This phytoestrogen was first isolated and identified in 1990 and is believed to exert several favored biological characteristics, including antiosteoporosis, antidepression, anti-inflammatory, antioxidant, and antitumor activities [81][82]. The modulation of various signaling pathways such as MiR-223-3p/ NALP3, IGF-1, TLR4/ NF-κB, PI3K/Akt, NFκB/ NALP3, Wnt1/ β-catenin, and Nrf-2 are documented as the basic mechanisms by which icariin possess its pharmacological and therapeutical functions [77].

The inhibition of interleukin-1β (IL-1 β)/ TGF-β-mediated activation of renal fibroblasts is the mechanism involved in the attenuation of renal fibrosis in chronic renal disease by icariin [83]. Furthermore, icariin can suppress cystitis induced by cyclophosphamide chemotherapy by the upregulation of the Nrf-2/HO-1 signaling pathway as well as the downregulation of the NF-кB pathway [84]. The neuroprotective characteristics of this phytochemical against Alzheimer's and Parkinson's diseases are mediated by affecting several biomolecules and molecular pathways such as amyloid precursor protein, β-site amyloid precursor protein cleaving enzyme 1 (BACE1), insulin-degrading enzyme, ERK1/2, GSK-3, NF-κB, Nrf2, and PI3K [80]. The inhibition of myocardial apoptosis, the prevention of inflammation on endothelial cells, the improvement of the immune system function, and the activation of HO1/Nrf2 signaling pathways are reported as the modulatory mechanisms by which icariin exerts its therapeutical properties against cardiovascular disorders [85][86]. Furthermore, the antimicrobial function of this phytoestrogen, such as ameliorating Escherichia coli lipopolysaccharide-mediated endometritis, is suggested to be performed by inhibiting oxidative stress and inflammation [87]. In addition, the desired effects of icariin on the skeletal system, such as the alleviation of osteoarthritis and inhibition of RANKL-induced osteoclast genesis, are mediated by the regulatory role on the autophagy of chondrocytes, modification of PI3K/AKT/mTOR signaling, inhibition of reactive oxygen species (ROS) levels, and reduction in the expression of *NOX1* and *NOX4* [88][89]. Moreover, the immunoregulatory and anti-inflammatory properties of icariin have introduced this phytochemical as a novel promising medicament to confront disorders related to the immune system, including inflammatory bowel diseases, asthma, multiple sclerosis, rheumatoid arthritis, lupus nephritis, atherosclerosis, and cancer via the restoration of aberrant signaling pathways, modulation of the functions and activation of immune cells, and regulation of the release of inflammatory factors [90].

Many studies have demonstrated the therapeutic function of icariin on several types of cancers, each of which was achieved by affecting a variety of cellular regulatory mechanisms. The amelioration of benign prostatic hyperplasia is demonstrated, which was achieved through the activation of the AMPK pathway as well as its antiproliferative (revealed histological manifestations), pro-apoptotic (upregulated *Bax* and downregulated *Bcl-2*), antioxidative (reduced malondialdehyde, catalase exhaustion, and decreased glutathione depletion), and anti-inflammatory (reduced IL-6 and tumor necrosis factor [TNF]-α levels) properties [91]. Moreover, icariin-induced upregulation of miR-7 expression and subsequent inhibition of PI3K/AKT and Raf1/ERK1/2 signaling pathways leads to suppression of benign prostatic hyperplasia cells proliferation, migration, and promotion of apoptosis [92]. The inhibition of SIRT6/NF-κB by icariin cause redox-mediated apoptosis in triple-negative breast cancer cells [93]. Furthermore, the suppression of autophagy and the regulation of the MELK-mediated PI3K/Akt signaling pathway are recognized as another mechanism by which icariin induces apoptosis in MCF-7 breast cancer cells [94][95]. Modification of the mTOR/PI3K/Akt signaling pathway by icariin leads to both apoptosis and autophagy and, finally, inhibition of the growth of human cervical cancer cells [96]. The reduction of TLR4/MyD88/NF-κB and Wnt/β-catenin pathways upon icariin administration leads to the alleviation of cervical cancer [97]. In lung cancer, it is demonstrated that icariin is able to target the miR‑205‑5p/PTEN axis leading to the modulation of the PI3K/Akt signaling pathway and inhibition of tumor progression [98]. The activation of the mitochondrial apoptotic pathway is reported as another mechanism that enables icariin to treat lung cancer [99]. In addition, the therapeutic effects of icariin on other types of cancer such as gastric, pancreatic, colon, and human oral squamous cell carcinoma are reported [100][101][102][103].

## Therapeutic Properties of Icariin on Ovarian Cancer

Similar to the other types of cancer mentioned earlier, icariin can prevent the proliferation and progression of ovarian cancer. Indeed, a multi-dimensional spectrum-effect relationship study, a scientific method based on the fingerprint of traditional Chinese medicines, determines the correlations between fingerprint and activity and proposes the antitumor activity of icariin against ovarian cancer [104]. Furthermore, a study based on network pharmacology suggested that icariin can target a variety of signaling biomolecules such as MMP-9, PIK3CA, STAT3, TNF, ERBB2, PIK3CA, mTOR, KDR, IL-2, and F2 in ovarian cancer SKOV3 cell line all of which leading to the induction of apoptosis and inhibition of proliferation through the suppression of PI3K/Akt signaling pathway [105]. Similarly, a most recent network pharmacology-directed experimental investigation demonstrated that in SKOV3 cells, icariin could induce apoptosis via affecting pro-apoptotic markers, including Bcl-xL, Bax, and caspase-3 as well as disrupting the activation of the NF-κB pathway and modulation of PI3K/Akt pathway [106].

In vitro studies have revealed that the functions of icariin on ovarian cancer cell lines are achieved through the modulation of autophagy and the promotion of apoptosis mediated by several signaling pathways [107]. In ovarian cancer A2780 cells, icariin downregulated the miR-21 expression, upregulated PTEN and RECK protein expression, and reduced pro-apoptotic Bcl-2 protein levels suggesting the regulatory role of icariin on ovarian cancer cells proliferation, apoptosis by modification of miR-21 expression, and the mentioned downstream proteins [107]. Furthermore, increased levels of apoptosis, higher levels of ROS, and altered cell cycle have resulted after the administration of icariin on OVCAR-3 ovarian cancer cells suggesting the cytotoxicity and apoptosis of this phytochemical against ovarian cancer cells [108]. Similar results regarding the cytotoxicity of icariin against ovarian cancer cells have been reported in SKOV-3 cells [109]. Furthermore, a recent study stated that the inhibition of proliferation, the stalled cell cycle, and the promotion of apoptosis via disruption of the TNKS2/Wnt/ β-catenin pathway mediated by the upregulation of *miR-1-3p* could be achieved after treatment of ovarian SKOV-3 cells with icariin [110].

In addition to the typical ovarian tumors, icariin can be considered a good choice for phenotypes that are more difficult to respond to and/or do not respond to the current chemotherapeutic strategies in the clinic. In the multidrug-resistant phenotype of SKVCR cells, for example, Jiang *et al*. revealed that icariin could activate the mTOR signaling pathway, followed by autophagy inhibition, apoptosis promotion, and suppression of ovarian cancer cell proliferation and tumorigenesis [111]. Also, these findings suggest that the antitumor activity of icariin represents a solution for multidrug-resistance types of ovarian cancer [111]. In addition, icariin could enhance the chemosensitivity of a common chemotherapeutic (cisplatin)-resistant phenotype of SKVCR cells via the inhibition of autophagy, induction of apoptosis, promoting G1/S cell cycle transition, and activation of the Akt/mTOR/ATG5 pathway [112].

## Conclusion

The current study revealed that reviewed investigations suggest promising therapeutical properties of icariin against ovarian cancer, which resulted from the regulatory role of this phytochemical on different signaling pathways determining the proliferation and growth or apoptosis and death of tumoral cells. Nevertheless, the current knowledge is limited to cellular studies. Hence, further experimental and clinical investigations are crucially required to assess the final safety and efficacy of icariin.

## Conflict of Interest

The authors declared that have no conflict of interest.
